# Environmental Nitrogen Losses from Commercial Crop Production Systems in the Suwannee River Basin of Florida

**DOI:** 10.1371/journal.pone.0167558

**Published:** 2016-12-01

**Authors:** Rishi Prasad, George J. Hochmuth

**Affiliations:** Soil and Water Science Department., Institute of Food and Agricultural Sciences, University of Florida, Gainesville, FL, United States of America; University of Waterloo, CANADA

## Abstract

The springs and the Suwannee river of northern Florida in Middle Suwanee River Basin (MSRB) are among several examples in this planet that have shown a temporal trend of increasing nitrate concentration primarily due to the impacts of non-point sources such as agriculture. The rate of nitrate increase in the river as documented by Ham and Hatzell (1996) was 0.02 mg N L^-1^ y^-1^. Best management practices (BMPs) for nutrients were adopted by the commercial farms in the MSRB region to reduce the amounts of pollutants entering the water bodies, however the effectiveness of BMPs remains a topic of interest and discussion among the researchers, environmental administrators and policy makers about the loads of nitrogen entering into groundwater and river systems. Through this study, an initiative was taken to estimate nitrogen losses into the environment from commercial production systems of row and vegetable crops that had adopted BMPs and were under a presumption of compliance with state water quality standards. Nitrogen mass budget was constructed by quantifying the N sources and sinks for three crops (potato (*Solanum tuberosum L*.), sweet corn (*Zea mays L*.) and silage corn (Zea mays L.)) over a four year period (2010–2013) on a large representative commercial farm in northern Florida. Fertilizer N was found to be the primary N input and represented 98.0 ± 1.4, 91.0 ± 13.9, 78.0 ± 17.3% of the total N input for potato, sweet corn, and silage corn, respectively. Average crop N uptake represented 55.5%, 60.5%, and 65.2% of the mean total input N whereas average mineral N left in top 0.3 m soil layer at harvest represented 9.1%, 4.5%, and 2.6% of the mean total input N. Mean environmental N losses represented 35.3%, 34.3%, and 32.7% of the mean total input N for potato, sweet corn, and silage corn, respectively. Nitrogen losses showed a linear trend with increase in N inputs. Although, there is no quick fix for controlling N losses from crop production in MSRB, the strategies to reduce N losses must focus on managing the crop residues, using recommended fertilizer rates, and avoiding late-season application of nitrogen.

## Introduction

Nitrogen is an important agricultural input and critical for crop production. However the ramifications of agricultural intensification for increasing food production have led to a cascade of environmental and human health problems, and will likely continue due to increasing human population [1–2]. Groundwater pollution from agricultural nitrate-nitrogen (NO_3_-N) loading is a worldwide problem [3–5]. When NO_3_-N is introduced to surface water from runoff or from groundwater discharges and subsequently to coastal ecosystems, it can promote eutrophication as well as increase populations of aquatic nuisance plants leading to an overall deterioration of the ecosystem [6–7].

The Suwanee River is the State River of Florida and is a major aquatic resource that begins in Georgia and flows through northern Florida before draining into the eastern Gulf of Mexico. The river was designated as an Outstanding Florida Water (OFW) in 1979 by the Florida Legislature. The Suwanee River Basin (SRB) in Florida covers 10,955 km^2^ [8] and contains the highest concentration of first magnitude freshwater springs (a spring that discharges at least 2.8 cubic meter of water per second) in the world. These springs are known for their aesthetic values drawing millions of tourists every year. Increasing incidences of algal bloom in the springs and river in the SRB during the last decade have focused the state’s attention on pollution preventive measures. Studies conducted in the SRB have shown an increasing temporal trend of NO_3_-N concentrations in the river and associated spring waters [7, 9, 10]. The average NO_3_-N levels in the springs in this basin have increased from 0.1 to 5 mg L^-1^ over a period of 40 years [11]. Similarly the river’s baseline annual median NO_3_-N concentration of 0.50 mg N L^-1^ in 1979 increased to 0.72 mg N L^-1^ by 2005 [10].

The agricultural industry in SRB has been economically important for many generations, producing a wide variety of agricultural products. With improvement in irrigation technologies, the irrigated acreage in the basin has significantly increased withdrawing groundwater from the Floridian aquifer system as the primary source of water [12]. Expansion in irrigated acreage coupled with increasing fertilizer use in the basin has been associated with increasing NO_3_-N concentrations in the water bodies [10–11]. Further, the soils in the agricultural production area of the basin are sandy, well drained to excessively drained, and belong primarily to soil orders Entisols and Ultisols which are vulnerable to nutrient leaching especially under excessive rainfall or irrigation conditions [13].

With the passage of the Federal Clean Water Act (FCWA) in 1972, states were required to assess the impacts of non-point sources of pollution on surface waters, and establish programs to minimize pollution through the establishment of Total Maximum Daily Loads (TMDLs). Total Maximum Daily Loads are defined as the maximum amount of a pollutant that a water body can receive and still meet the water quality standards as established by states through the 1972 Clean Water Act. In response to meeting the TMDL requirements, water quality protection programs such as best management practices (BMP) were developed for pollutants entering the water bodies from non-point agricultural sources. Best management practices are defined as the practices or combinations of practices determined by research or field testing in representative sites to be the most effective and practicable methods of fertilization designed to meet nitrate groundwater quality standards, including economic and technological considerations (Florida Statutes Chapter 576). The BMPs were adopted as rules by Florida legislature and implemented as incentive based voluntary programs. The growers who implemented a BMP program were considered to be operating under a presumption of compliance with state water quality standards [14].

Many progressive farms in SRB adopted the BMPs and implemented nutrient management plans with the assistance of major state and federal agencies and the University of Florida. However the effectiveness of the BMPs remains a topic of interest and an ongoing discussion among researchers, environmental administrators and policy makers [15]. There is a need to investigate and evaluate the N losses from commercial farms that had adopted a BMP program. In this regard, nitrogen budgets can be of significant importance for understanding the sources and fates of N at the field, farm, or watershed levels. A nitrogen budget is defined as the summary table of the book-keeping of nutrient inputs and outputs of a system [16]. Nitrogen budgets can help identify and describe imports, recycling pools and exports of N, and can also help to identify any N build up area or vulnerable area prone to N losses. Upon quantification of N pools, BMPs can be targeted or further refined to reduce N losses from agricultural systems. Liu et al. [17] used the N budget to investigate N losses in a winter wheat- maize cropping system in a clay loam soil and found leaching of NO_3_-N was the primary pathway for N loss. Similarly, Kraft and Sites [4] used the N budget to estimate NO_3_-N loading rates from irrigated sweet corn and potato fields in the Central Sand Plains area of Wisconsin.

Through this study, an initiative was taken to estimate nitrogen losses into the environment from commercial production systems of row and vegetable crops that had adopted BMPs and were under a presumption of compliance with state water quality standards. The main objectives of this study were: 1) to quantify the major sources (inputs) and sinks (outputs) of N on a typical commercial vegetable (potato and sweet corn) and row crop (silage corn) production system of Middle Suwanee River Basin (MSRB) in Florida over a four-year study period; 2) compare the potential environmental N losses, N exports and N cycling from three aforementioned crops over four year study period

## Materials and Methods

### Study Site, Regional Geology and Weather

This study was conducted at the request and approval of the farm owner. No permits or approvals from any state or federal regulatory agency were required. George Hochmuth can be contacted for more information and any future permissions. The study site was a commercial farm located in MSRB at O’Brien, in Suwannee County, Florida (latitude 30.04 and longitude -82.94).The Suwannee River is 1.2 km south-west of the farm which is part of a large area of poultry, dairy, and crop agriculture. Earlier studies carried out by Hornsby [10] and Pitman et al. [18] indicated an occurrence of high N loads in the MSRB. The direction of local groundwater flow was south to southwestward from the agricultural area towards the Suwanee River as documented in potentiometric surface maps for years 2002, 2005, and 2009 [19].The farm has 42 individual center pivot irrigated fields (of average size 55 ha), spread across a land area of 2020 ha, and has a confined beef cattle feeding management system, along with anaerobic digester for waste management [20]. Main crops grown on the farm are potato, sweet corn, silage corn, peanut, cotton, and several other vegetable crops. The confined animal management system consists of five barns where approximately 5000 head of beef cattle are feed. The waste (mixture of manure and bedding materials (peanut hulls, old hay, and saw dust)) collected from the barns serve as a feedstock for the digester unit. The digester unit is a two-stage mixed plug-flow anaerobic digester and generates solid and liquid effluent along with biogas. The solid and liquid effluents are used for land application in silage corn fields.

**Fig 1:**
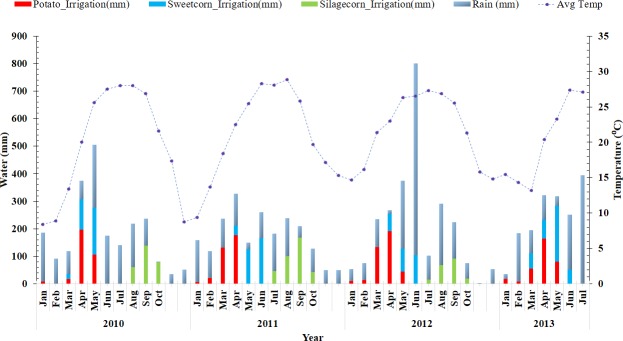
Average monthly temperatures, monthly total rainfall, and monthly total irrigation applied to potato, sweetcorn and silage corn during the growing season between 2010 and 2013 at the study farm located in Middle Suwannee River Basin, Florida.

The regional location of the farm is characterized by presence of karst features such as sinkholes, springs, solution conduits, etc., and highly permeable sands atop the upper Floridian aquifer allowing the opportunity for direct hydraulic and geochemical interactions between surface water and groundwater [21]. Information on regional geology, climate and soils can be found elsewhere [22]. Briefly, the farm lies along a physiographic region of Cody Escarpment where the sands are thick and the eroded Hawthrone formation consist of thin and pocketed clay to no clay atop the limestone. The geological features of Cody Escarpment and eroded Hawthorne formation increase the threats to water quality of Suwannee River. The climate of the Suwannee River Basin is a mixture of warm temperate and subtropical conditions with mean annual temperature of 20.3°C and annual precipitation averaging about 1356 mm. Thunderstorms are more pronounced in summer months (June through September). More information on weather conditions at the farm are presented in Fig 1. The soils in the agricultural areas belong primarily to Entisols or Ultisols where texture in the root zone is usually sand or fine sand regardless of the soil order [13]. The soils at the study site are Alpin fine sand (Thermic, coated lamellic, Quartzipsamments) and belong to hydrologic group A, making irrigation critical for the economic viability of the farming operation.

### Nitrogen Budget and Calculation of Environmental Nitrogen Loss

A Nitrogen budget is based on the concept of mass conservation (see Eqs 1–3) and is used for accounting major inputs and outputs of N for a defined systems for a defined time period [22–24]. For this study, nitrogen budgets were prepared for potato, sweet corn, and silage corn for the growing seasons between 2010 and 2013. Potato and sweet corn were grown as spring season crops whereas silage corn was grown as fall season crop. Directly measured N inputs in this study were 1) initial or baseline mineral N (N_solin_) (1M KCl extractable NH4-N+NO3-N) present in the soil tillage zone (0.3m soil depth) before planting, 2) nitrogen from fertilizer (N_fert_), 3) mineralizable-N from application of digester effluents (Nefflu), and 4) nitrogen from wet atmospheric deposition (N_atm_). Nitrogen inputs that had higher uncertainty associated with their measurement were not accounted in the budget and justifications for doing so were addressed by Oenema et al [16]. We did not account N inputs of seed (N_seed_), net N mineralization (mineralization-immobilization) of soil organic matter (N_netmin_), and N in irrigation water (N_irr_) as their N content was negligible and suffered from greater spatial, temporal, and measurement variations compared to other N pools. For example, the average soil organic N in the plough layer (0.3m) was 0.03% and the average soil organic matter was 0.73%. Assuming a mineralization factor of 2%, the estimated annual N mineralization would be 6 kg N ha^-1^ yr^-1^ or 2 kg N ha^-1^ season^-1^ (the average length of crop season was four months). The directly measured N outputs in the budget were 1) crop N uptake (N_crop_), and 2) mineral N left in the soil at crop harvest (N_solfi_). The difference between the measured N inputs and outputs of the nitrogen budget was considered as “unaccounted-for N” and used as a metric for estimation of “seasonal environmental N losses (N_envloss_)” (Eq 6). The N_envloss_ consisted of leaching and gaseous losses of N (via volatilization and denitrification pathways) (Eq 4). Surface runoff loss was not observed in the study fields due to the flat topography and sandy soils, hence was excluded from the budget. The N mass balance equations describing the components of the nitrogen budget are as follows:

$$\sum N_{inputs}=\sum N_{outputs}$$ (1)

Where,

$$N_{inputs}=N_{solin}+N_{fert}+N_{efflu}+N_{seed}+N_{atm}+N_{irr}+N_{netmin}$$ (2)

$$N_{outputs}=N_{crop}+N_{solfi}+N_{leach}+N_{vol}+N_{den}$$ (3)

$$N_{envloss}=N_{leach}+N_{vol}+N_{den}$$ (4)

Ignoring N_seed_, N_irr_ and N_netmin_ in Eq 2 and replacing N_leach_ + N_vol_ + N_den_ with N_envloss_ in Eq 3, Eq 1 can be written as:

$$N_{solin}+N_{fert}+N_{efflu}+N_{atm}=N_{crop}+N_{solfi}+N_{envloss}$$ (5)

Rearranging Eq 5

$$N_{envloss}=N_{solin}+N_{fert}+N_{efflu}+N_{atm}-N_{crop}-N_{solfi}$$ (6)

Where, N_inputs_ refer to all the sources of N; N_outputs_ refer to all the sinks of N; N_solin_: initial or baseline mineral N; N_fert_: N from fertilizer; N_efflu_: N from digester effluent; N_seed_: N present in seed; N_atm_: N from atmospheric deposition; N_irr_: N in irrigation water; N_netmin_: N from net mineralization (mineralization—immobilization); N_crop_: N uptake by crops; N_solfi_: N left in the soil after crop harvest; N_leach_: N lost via leaching; N_vol_: N lost via volatilization; N_den_: N lost via denitrification; N_envloss_: N lost in environment or the unaccounted for N. All values were expressed in kg ha^-1^ season^-1^. The N_solfi_ can also become an environmental loss depending on the length of fallow period between the consecutive crops and rainfall amounts during the fallow period.

### Sampling Methods

#### Plant Sampling and Analysis

To construct nitrogen budgets, field methods focused on plant and soil sample collection at several locations (within individual fields) on the farm between growing seasons of 2010 and 2013. Fields and Pivots are used interchangeably hereafter. Nitrogen removal in the crop biomass was determined at crop physiological maturity on randomly selected pivots at the study farm. Information on crop management for the three crops is presented in Table 1. To adequately represent the conditions within each field, plants were sampled at twelve random locations in 1.5-m sections of the row, separated into individual plant parts and washed gently to remove sand particles. Potato plants were separated into tubers, shoots, and roots. Sweet corn and silage corn plants were separated into roots, stalk, leaves, ears (unhusked), and stubble (portion of the stalk (15 cm) left on the soil surface after mechanical harvesting). The plant parts for all crops were oven dried at 70°C for 48 to72 hours until constant dry weight. The dried plant parts for sweet corn and silage corn were shredded using a mechanical shredder followed by grinding in a Wiley mill and obtaining a subsample for lab analyses. The N determinations were made on the tissue samples using the Kjeldahl digestion followed by semi-automated colorimetry (EPA Method 351.2) using Technicon AAII (Technicon Instruments Corp., Tarrytown, NY, USA). Tissue nitrate-N was determined using 2N KCl extraction followed by semi-automated colorimetry (EPA Method 353.2). Negligible amounts (below method detection limit of 0.05 mg/l) of nitrate were detected in the tissue samples, therefore all plant tissue N determined following Kjeldahl digestion was considered as total N. Plant part dry matter N uptake per 1.5 m section of row was calculated by multiplying individual tissue N concentration (%) by respective plant part dry matter weights and divided by 100. Total crop dry matter N uptake was calculated as the sum of dry matter N in individual plants parts and expressed in kg ha^**-1**^ [24].

**Table 1:** Crop management information for potato, sweet corn, and silage corn grown at the study farm.

Crop	Year	Pivot ID	Planting date	Row spacing (m)	Plant spacing (m)	Harvest date	Total N (kg ha^-1^)	Total P (kg ha^-1^)	Total K (kg ha^-1^)
**Potato**^**#**^	2010	Pivot 19	10-Feb	1.01	0.1	20-May	265	47	380
	2011	Pivot 12	27 Jan-3 Feb	1.01	0.1	28-Apr	278	51	354
	2011	Pivot 17	11–18 Feb	1.01	0.1	20-May	285	51	358
	2012	Pivot 10	30 Jan-4 Feb	1.01	0.1	02-May	285	49	339
	2012	Pivot 18	18–25 Feb	1.01	0.1	20-May	248	49	337
	2013	Pivot 12	14–21 Feb	1.01	0.1	20-May	248	49	343
**Sweet corn**^**#**^	2010	Pivot 5	25 Feb-5 Mar	0.76	0.19	25-May	282	23	157
	2011	Pivot 4	28Apr- 4 May	0.76	0.19	28-Jun	319	21	262
	2011	Pivot 5	23–27 Apr	0.76	0.19	28-Jun	274	21	262
	2012	Pivot 3	30Apr-3 May	0.76	0.19	04-Jul	311	25	129
	2012	Pivot 22	25–28 Apr	0.76	0.19	28-Jun	304	25	121
	2013	Pivot 5	18–27 Mar	0.76	0.19	07-Jun	384	25	240
**Silage corn**^**#**^	2010	Pivot 12	30-Jul	0.76	0.17	18-Oct	172 +68*	23	131
	2011	Pivot 5	1–6 Aug	0.76	0.17	11-Oct	101+81*	25	236
	2011	Pivot 16	14–16 July	0.76	0.17	10-Oct	210+ 51*	25	236
	2012	Pivot 24	14–16 July	0.76	0.17	04-Oct	326	25	223
	2012	Pivot 25	12-Jul	0.76	0.17	04-Oct	315	25	226

* N from effluent

^#^Potato cultivar: Red La Soda; Sweet corn cultivars: Beyond yellow, Garrison yellow, Obsession bicolor, Accentuate yellow, 7143 bicolor; Silage corn cultivars: 1022, 2023, 99

#### Soil Sampling and Analysis

Two soil cores were removed close to the plant sampling area at all twelve locations within the fields. The soil was sampled at harvest to a depth of 0.3 m, in increments of 0.15 m, using 0.05-m-diameter metal probes. Pre-plant soil core samples were also collected to establish the baseline or initial soil mineral-N (Nsolin). The duplicate soil cores taken at each locations and depths were composited, air dried (40°C for 48 hours), sieved to pass 2-mm screen, and analyzed for 1 M KCl extractable nitrate and ammonium-N (soil: solution ratio of 1:10) per standard procedures [25]. Nitrate-nitrogen and ammonium-N determinations were made by automated colorimetric analysis (EPA method 353.2 and 350.1(modified) respectively) using the Alpkem Flow Solution IV (OI Analytical, College Station, TX, USA). Mineral N was estimated as sum of 1 M KCl extractable nitrate and ammonium-N. Soil extract concentrations were converted to kg ha^**−1**^ N, using soil moisture correction factor (determined from oven dry mass), bulk density values and soil depths. Measurements at twelve locations were averaged for each 0.3-m soil depths.

#### Mineralization Estimation from Digester Effluent

Since liquid effluent from the digester was surface-applied to silage corn fields, an estimate of N mineralization was required. To estimate the contribution of N (via mineralization) from surface application of liquid effluent, a separate in-situ column experiment was established at the University of Florida, Plant Science Research and Education Unit, in Citra, Florida [26]. From the study it was found that 78% (data not shown) of organic N present in the liquid effluent mineralized within 20 days after its application in the field [27]. Hence a mineralization factor of 0.78 was used to calculate short term mineral-N contribution from application of liquid effluent.

Liquid effluents were sampled at different times during the year and analyzed for total Kjeldahl-N, ammonium-N, and pH, among others, according to procedures recommended by Peters et al. [28]. The effluent application rates were obtained from the farm records and short term mineralization estimates were calculated by multiplying injection rate with the organic N concentrations sampled closest to the application date and the factor 0.78. The average organic N concentration of liquid effluent was 1430 mg L^-1^.

#### Atmospheric Deposition

Data on wet atmospheric deposition from rainfall for NO_**3**_-N and ammonium-N were obtained from closest National Atmospheric Deposition Program monitoring station located at the Branford site (rural area), Florida (FL03) [29], 16 km from the farm site. Wet deposition was calculated by multiplying the monthly estimates of precipitation-weighted mean ion concentrations (NO_**3**_-N + NH_**4**_^**+**^-N mg L^**-1**^) by total monthly rainfall (amounts in cm) and dividing by 10. Contribution of dry deposition was relatively minor (5% of wet deposition) as indicated by data from a nearest Clean Air Status and Trends Network site located at Sumatra (approximately 80 km from the farm), Florida (site ID: SUM156) [30]. Hence its contribution towards total atmospheric deposition was omitted from the budget.

#### Crop Management

All crop management decisions such as tillage, planting, irrigation, fertilization, pest and disease management, harvesting and other operations were undertaken by the cooperating farmer and the information pertinent to this study was provided by them as documented in their farm records. The management decisions at the study farm represented average grower practices in the MSRB (personnel communication with farmer). Important crop management information details pertinent to this study are presented in Table 1. Information on irrigation, monthly precipitation and average temperatures are presented in Fig 1.

#### Measurement of Nitrogen Leaching

With the objective of measuring nitrate leaching (N_**leach**_) (one of component of the environmental N losses) at the farm, eight drainage lysimeters [31] were installed at random locations within pivot-5 in December 2010.

At each lysimeter location, soil was excavated in the size of a rectangular reservoir (0.81 m x 0.58 m) (made by cutting a 208 L plastic drum into half lengthwise) and the basin of the lysimeter was installed 1.2 m deep from soil surface at an incline to facilitate the drainage of leachate in a sampling reservoir (19 L) via a leachate retrieval spout. The basin of the main reservoir was filled with 10 cm thick layer of pebbles and covered with a plastic screen (10^−6^ m^2^ pore size) to prevent mixing of soil and the pebbles and to allow free drainage of water into the sampling reservoir. The soil was replaced in its original horizon sequence and repacked. The sampling reservoir was accessed through a tygon tubing within a capped PVC pipe (retractable) which connected the sampling reservoir to soil surface. Leachate samples were collected from the sampling reservoir approximately every two weeks and more frequently after heavy rainfall events. The leachate was pumped manually using a ShopCraft Multi-Use Pump (Part NO. W1145, Advanced Auto Parts, Inc., Roanoke, VA, USA) and the total leachate volume was recorded. The sampling procedure and laboratory quality control requirements were carried out according to Environment Protection Agency certification guidelines [32]. Water samples were analyzed for NO_3_-N, ammonium-N and TKN by automated colorimetric analysis using EPA Method 353.2, EPA Method 350.1 and EPA Method 351.2 respectively. NO_3_-N and ammonium-N concentrations were determined using an Alpkem Flow Solution IV (OI Analytical, College Station, TX, USA) whereas TKN was analyzed using Astoria 2 Segmented Flow Analyzer (Astoria-Pacific, Inc., Clackamas, OR, USA). Ammonium-N and TKN concentrations were detected in few rare occasions and were insignificant; hence N leaching loss was estimated primarily from NO_3_-N concentrations. For each sampling event, total leachate volume was multiplied by the NO_3_-N concentrations and values scaled to a per-hectare basis to estimate the N leaching loss (kg ha^-1^ N). The leaching losses were added over each sampling location and sampling date to determine the cumulative seasonal N leaching loss.

The lysimeters suffered from breakages and did not allow collection of complete season data. It took nearly two years before the lysimeters stabilized and a complete one-season leaching data were obtained for sweet corn planted in pivot 5 in spring 2013.

Additionally, 2013 spring season provided a unique scenario since the farmer applied liquid effluent N before planting sweet corn in pivot 5. The liquid effluent was applied between 12th and 14th March, 2013. The estimated mineralizable N from effluent application was 121 kg ha^-1^ N. Sweet corn was planted between 18^th^ and 27^th^ March, 2013, and harvested on 10^th^ June, 2013 (84 days after planting). The farmer applied 311 kg ha^-1^ synthetic fertilizer-N in 8 split applications during the growing season

The lysimeters were pumped out to empty on 4^th^ March, 2013 (to establish the baseline), and sampled thereafter every two weeks until crop harvest on 7^th^ June, 2013 (three days before farmers harvest date). Plants were sampled close to lysimeters locations and dry matter and tissue N determinations were made as described in the plant sampling section.

Soil cores were also collected at 0.3 m increments to a soil depth of 1.2 m in close vicinity of lysimeters on both the dates (4^th^ March and 7^th^ June, 2013). The soil cores for individual depths were air dried and analyzed for NO_3_-N and ammonium-N according to procedures described in soil sampling section and expressed in units of kg ha^-1^N. The mineral N content of 1.2 m soil profile was calculated by adding the NO_3_-N and ammonium-N concentrations for respective soil depths and averaged across all lysimeters locations.

Nitrogen lost as gas (N_gas_) was calculated as follows:

$$N_{solin}+N_{fert}+N_{efflu}+N_{atm}=N_{crop}+N_{solfi}+N_{leach}+N_{vol}+N_{den}$$ (7)

$$N_{gas}=N_{vol}+N_{den}$$ (8)

Substituting Eq 8 in Eq 7 and rearranging Eq 7:

$$N_{gas}=N_{solin}+N_{fert}+N_{efflu}+N_{atm}-N_{crop}-N_{solfi}-N_{leach}$$ (9)

Where,

N_gas_: N lost as gas via volatilization and denitrification

All units in kg ha^-1^season^-1^ N and defined previously.

### Statistical Analysis

Analysis of variance was conducted using the Proc GLM in SAS^®^ 9.3 [33] to study the effect of year, crop, interaction effect of year and crop, field, and location within field on response variable using the following model:

$$Y_{ijkl}=µ+\zeta_{i}+\beta_{j}+{\left(\zeta \beta \right)}_{ij}+\epsilon_{ijk}+\delta_{ijkl}\;\mathrm{and}\;\epsilon_{ijk}\sim^{iid}N\left(0,\sigma_{\epsilon }^{2}\right);\delta_{ijkl}\sim^{iid}N\left(0,\sigma_{\delta }^{2}\right)$$ (10)

Where, Y_ijkl_ is the response variable in Year i, Crop j, Field k and Location l, and where i = 1,…4, j = 1,2,3, k = 1,…n_ij_, l = 1,…12; μ is the overall mean, ζ_i_ is the main effect of i^th^ year, β_j_ is the main effect of j^th^ crop, (ζβ)_ij_ is the interaction between i^th^ year and j^th^ crop, ∈_ijk_ is field random effects and δ_ijkl_ is the sampling location error. The Tukey-Kramer adjusted P values were used to determine differences in means at α = 0.05 significance level. The assumptions of homogeneity of variance and normality of residuals were checked for the final models.

## Results and Discussion

### Nitrogen Inputs

Nitrogen inputs to potato, sweet corn, and silage corn crops during their individual growing seasons are presented in Table 2. Nitrogen inputs varied between crops and years. On average and within one standard deviation (±SD), potato, sweet corn, and silage corn received N inputs of 274.5 ± 17.0, 331.5 ± 46.5 and, 265.7 ± 53.3 kg ha^-1^, respectively. Fertilizer N was the largest, primary N input and represented 98.0 ± 1.4%, 91.0 ± 13.9%, 78.03 ± 17.3% of the total N input for potato, sweet corn, and silage corn, respectively. All fertilizer N rates applied by the grower were above the university (UF/IFAS) recommended rates (Table 1). The university recommended fertilizer N rates are 224, 224 and 235 kg ha^-1^ for potato, sweet corn, and silage corn, respectively [34–35]. In other studies, such as Kraft and Sites [4] reported typical fertilizer N applications of 258, 200 and 180 kg ha^-1^ N for potato, sweet corn and field corn respectively in sandy soils of Wisconsin Central Sand Plain. Liquid effluent N applied to silage corn fields during 2010 and 2011 growing season represented 30 ± 11% of the total N input. The baseline soil mineral-N amounts were variable among fields and represented 2.5 ± 0.4, 6.0 ± 2.0, and 6.0 ± 1.0% of the total N input for potato, sweet corn, and silage corn, respectively. The average baseline mineral N was 6.8 ± 3.3, 18.4 ± 9.9, 19.2 ± 19.4 kg ha^-1^ N for the three mentioned crops. Presence of smaller amounts of baseline mineral N in plough layer clearly indicated residual N from previous crop had moved down to lower soil profile. Contribution of wet atmospheric deposition was minor (1.0 kg ha^-1^ N) relative to other N inputs during the growing seasons. Li et al. [36] reported 4 kg ha^-1^ year^-1^ as the annual atmospheric deposition rate of N in Florida.

**Table 2:** Nitrogen budgets for potato, sweetcorn and silage corn grown at the study farm during the period 2010 to 2013. The abbreviations in the table can be referred to Eqs 1 through 9 in materials and methods section.

			Inputs					outputs			Unaccounted
			kg ha^-1^ season^-1^								∑ N_input_ -∑N_output_
**Crop**	**Year**	**Pivot ID**	**N**_**solin**_	**N**_**fert**_	**N**_**efflu**_	**N**_**atm**_	**∑ N**_**input**_	**N**_**crop**_	**N**_**solfi**_	**∑N**_**output**_	**N**_**envloss**_
**Potato**	2010	Pivot 19	Neg	265	‡ N/A	1	266	†175 ± 29	7± 0	182 ± 29	84 ± 29
	2011	Pivot 12	7 ± 2	278	N/A	1	285 ± 3	115 ± 20	30 ± 24	145 ± 31	140 ± 31
	2011	Pivot 17	Neg	285	N/A	1	286	158 ± 35	19 ± 8	178 ± 33	108 ± 33
	2012	Pivot 10	10 ±3	285	N/A	1	296 ± 3	181 ± 36	18 ± 12	200 ±42	96 ± 43
	2012	Pivot 18	7 ± 2	248	N/A	1	256 ± 2	165 ± 38	47 ± 35	212 ± 64	44 ± 64
	2013	Pivot 12	3 ± 2	248	N/A	2	253 ± 2	142 ± 22	10 ± 7	152 ± 28	101 ± 28
**Mean ± SD**			**7 ± 3**	**269 ± 17**		**1 ± 0.4**	**275 ± 17**	**154 ± 37**	**24 ± 23**	**178 ± 47**	**97 ± 50**
**Sweet corn**	2010	Pivot 5	Neg	282	N/A	1	283	185 **±** 11	10 **±**0	194 **±** 11	89 **±** 11
	2011	Pivot 4	Neg	319	N/A	1	320	214 **±** 31	21 **±** 7	235 **±** 29	85 **±** 29
	2011	Pivot 5	7 **±** 1	274	N/A	1	282 **±** 1	199 **±** 17	12 **±** 3	211 **±** 17	71 **±** 17
	2012	Pivot 3	22 **±** 27	311	N/A	1	334 **±** 27	167 **±** 33	19 **±** 6	187 **±** 36	148 **±** 35
	2012	Pivot 22	21 **±** 8	304	N/A	1	326 **±** 8	176 **±** 31	12 **±** 6	188 **±** 35	138 **±** 31
	2013^§^	Pivot 5									
**Mean ± SD**			**20 ± 17**	**293 ± 21**	**121**	**1**	**332 ± 46**	**203 ± 43**	**15 ± 6**	**218 ± 44**	**114 ± 42**
**Silage corn**	2010	Pivot 12	Neg	172	68	1	241	159 **±** 30	7 **±** 3	166 **±** 31	75 **±** 31
	2011	Pivot 5	12 **±** 3	101	81	1	195 **±** 3	156 **±** 32	8 **±** 3	164 **±** 33	30 **±** 32
	2011	Pivot 16	Neg	211	50	1	262	151 **±** 18	9 **±** 2	160 **±** 18	102 **±** 18
	2012	Pivot 24	27 **±** 32	326	0	1	354 **±** 32	197 **±** 29	7 **±** 3	205 **±** 30	150 **±** 51
	2012	Pivot 25	18 **±** 7	315	0	1	334 **±** 7	226 **±** 33	5 **±** 1	231 **±** 33	103 **±**38
**Mean ± SD**	** **	** **	**19 ± 19**	**208 ± 75**	**49 ± 31**	**1**	**266 ± 53**	**172 ± 39**	**7 ± 3**	**179 ± 38**	**87 ± 47**

† Standard deviation (SD).

‡ Not applicable.

§ The budget is presented in Table 3.

Neg refers to negligible amounts of nitrogen.

### Nitrogen Outputs

Nitrogen outputs for potato, sweet corn, and silage corn crops are presented in Table 2. Average crop N uptake represented 55.5%, 60.5%, and 65.2% of the mean total input N ranging from 115.2 to 181.1, 167.3 to 264.1, and 150.8 to 225.8 kg ha^-1^ N, for potato, sweet corn, and silage corn, respectively. These N uptake represented an average yield of 35 ± 4 Mg/ha (fresh), 23 ±5 Mg/ha (fresh), and 14 ± 2 Mg/ha (drymatter) for potato, sweet corn and silage corn respectively. Average mineral N left in top 0.3 m soil layer represented 9.1%, 4.5%, and 2.6% of the mean total input N and ranged from 7.0 to 46.8, 9.5 to 20.9, and 5.5 to 9.0 kg ha^-1^ N for potato, sweet corn, and silage corn, respectively. Similar estimates of crop N uptake have been reported in literature. For example, Errebhi et al. [37] reported N uptake in potato grown in sandy loam soils ranged from 78 to 185 kg ha^-1^ N, with fertilizer N applied at 270 kg ha^-1^. They reported average N recoveries of 33% (of the applied N) during a high leaching year and an average recovery of 56% during a season with fewer leaching events. Andraski and Bundy [38] studied N recoveries in potato and sweet corn grown in Plainfield sands using ^15^ N-depleted ammonium nitrate applied at 224 and 190 kg ha^-1^ N. They found that the total N uptake in potato and sweet corn was 139 and 186 kg ha^-1^ N, respectively. However only 50 and 65% N came from labelled fertilizer while the remaining N came from soil, water, and unknown sources.

**Fig 2:**
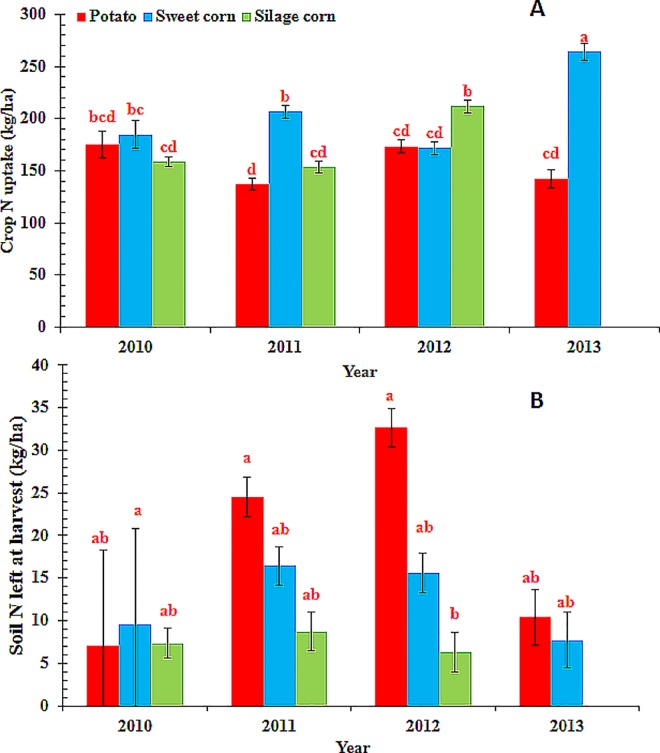
**Comparison of A) Nitrogen uptake (N_crop_) and B) mineral N remaining in soil after crop harvest (Nsolfi) between three crops (potato, sweet corn and silage corn) and four growing seasons (2010to 2013) at the study farm in the Middle Suwannee River Basin, Florida.** Silage corn was studied only between 2010 and 2012. Mean values of N_crop_ and N_soilfi_ (represented by individual bars and their standard errors for the three crops) followed by different letters indicate significant difference at α = 0.05 level.

Crop N uptake differed among crops (P < 0.0001) and year (P< 0.002). The interaction effect of crop and year was also significant (P < 0.02) and the means are presented in Fig 2A. Nitrogen uptake by silage corn and sweet corn differed between years whereas no such difference was found in case of potato (Fig 2A). Nitrogen uptake in sweet corn was greater in 2013 than the other years. Nitrogen uptake in silage corn was greater in 2012 than the other years. Comparisons of N uptake between crops during a year indicated greater N uptake by sweet corn than potato and silage corn in 2011, whereas more N accumulation occurred in silage corn than potato and sweet corn in 2012 (Fig 2A). The differences in crop N uptake between crops and years might be due to the differences in N inputs, management related factors (such as planting dates, fertilizer application timing, irrigation amounts etc.) and weather conditions.

Soil mineral-N remaining after crop harvest represented a potential leachable N source in the absence of an immediate deep rooted cover crop and was significantly different between years (P< 0.002) but not crops (P = 0.12). However, the interaction effect of year and crop was also found significant (P = 0.02) (Fig 2B). There were no significant differences in soil mineral N between years for crops except for 2012 when a greater amount of mineral-N remained in the soil in potato fields after crop harvest. The farmer applied 40 kg ha^-1^ fertilizer-N late in the growing season in response to a heavy rainfall event (hurricane Debby). The fertilizer application timing was close to plant physiological maturity, hence a greater amount of soil mineral-N was not used by the crop and remained in the soil in potato fields after crop harvest. Errebhi et al. [39] reported that soil NO_3_-N left at harvest increased quadratically as the proportion of N applied at planting increased. Soil mineral N left in the field after crop harvest was highly variable. Greater amounts of mineral N in the soil plough layer at harvest was indicative of late season fertilizer application, whereas smaller amounts remaining at harvest indicated movement of N to lower soil profiles which could not be captured by plant roots. In either cases N was highly susceptible to leaching loss that would lead to groundwater contamination.

### Environmental Nitrogen Losses

**Fig 3:**
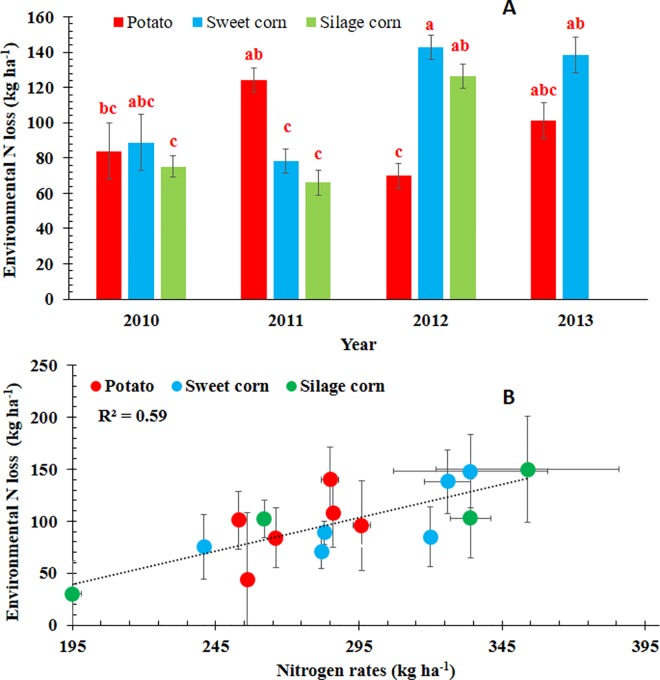
**Comparison of A) environmental nitrogen losses (N**_**envloss**_**) from three crops (potato, sweet corn and silage corn) during four growing seasons (2010 to2013) and B) relationship between seasonal total N rates and environmental nitrogen losses at the study farm in the Middle Suwannee River Basin, Florida.** Silage corn was not studied during 2013. Mean values of N_envloss_ (represented by individual bars and their standard errors for the three crops) followed by different letters indicate significant difference at α = 0.05 level.

The unaccounted-for N in the N-budget was used as an estimate of the amount of N lost from the crop production systems, and referred herein as environmental N losses (N_envloss_). Main effect of year on N_envloss_ was not significant (P = 0.25) whereas main effect of crop (< .0001) and interaction effect of year and crop were found significant (P<0.001) (Fig 3A). Mean N_envloss_ represented 35.3%, 34.3%, and 32.7% of the mean total input N for potato, sweet corn, and silage corn, respectively. A linear relationship was also observed between the N rates and the environmental nitrogen losses indicating greater amounts of N was lost with increasing amounts of N inputs (r^2^ = 0.59) (Fig 3B). During the study period, mean N_envloss_ ranged from 43.7 to 140.1, 71.4 to 147.7, and 30.4 to 149.8 kg ha^-1^season^-1^N, for potato, sweet corn, and silage corn, respectively (Table 2). Similar estimates of N losses have been reported by other researchers under different settings. For example, Kraft and Sites [4] reported NO_3_-N losses of 228 kg ha^-1^ from potato fields and 126 to 169 kg ha^-1^ from sweet corn fields in sandy soil in Wisconsin Central Sand Plain. Errebhi et al. [37]) reported NO_3_-N leaching of 71 to 257 kg ha^-1^ from potato grown in loamy sand plots fertilized with 270 kg ha^-1^ N.

Environmental N losses from potato fields was similar across all years except 2012. Sweet corn fields presented a similar case as potato fields except year 2011 (Fig 3A). The N_envloss_ from silage corn fields however presented a different scenario than potato and sweet corn fields (Fig 3A). Greater amounts of N were lost from silage corn fields in 2012 than 2010 and 2011. During 2012, the silage corn fields received all N from synthetic fertilizer compared to 2010 and 2011 when the fields received N from both fertilizer and liquid effluent sources. It appeared that faster solubility of nitrate-N from synthetic fertilizer sources made N susceptible to losses compared to liquid effluent which had to go through the mineralization process before making the N was available for plant uptake or losses.

Comparisons of N_envloss_ between crops within a year indicated no significant difference in N losses during 2010 and 2013 (Fig 3A). However, in 2011, more N was lost from potato fields than sweet corn and silage corn compared to year 2012 (Fig 3A). The shallow root system of potato is often considered as one of the major cause for poor N use efficiency by the potato crop alongwith with poor water and nutrient holding capacity of sandy soils [40]. Errebhi et al. [37]) reported linear increases in N losses with proportionate increase in N supply from potato fields. A similar relationship was found by Kraft and Sites [4] for sweet corn. The greater N loss from potato in 2011 may also be related to the late-season application of N by the farmer in response to the Tropical Storm Debby rainfall. The farmer applied 40 kg ha^-1^N at 70 days after planting when the plant was in late tuber bulking period.

### Identification of Unaccounted for Nitrogen in 2013 Spring Sweet Corn

Sweet corn grown in 2013 spring season provided a unique opportunity to identify the various N loss pathways leading to the unaccounted-for N. A detailed N budget for the sweet corn grown in spring 2013 season was constructed and is presented in Table 3. The crop received an average of 454.3 ± 21.4 kg ha^-1^ N in inputs of which the contributions of N_fert_, N_efflu_, and Ns_olin_ were 57.9%, 26.7%, and 15.0%, respectively. The contributions of N_irr_ and N_atm_ were negligible (1.0 kg ha^-1^ N, each) compared to other N inputs. Crop N uptake represented an average of 57.3% of the input N whereas N_solfi_ and N_leach_ represented an average of 14.5 and 10.4% of the input N. The unaccounted-for N in the budget represented 18.1% of the input N.

**Table 3:** Nitrogen budget for 2013 spring sweet corn grown in pivot 5 at the study farm.

Nitrogen inputs	Value (kg ha^-1^ season^-1^)	Nitrogen outputs	Value (kg ha^-1^ season^-1^)
1. N_solin_	68 ± 21	1. N_crop_	260 ± 29
2. N_fert_	263	2. N_solfi_	66 ± 16
3. N_efflu_	121	3. N_leach_	47 ±24
4. N_irr_	1	4. **N**_**gas**_	
5. N_atm_	1	5. Fertilizer applied 3days before harvest	48
**Ʃ Input**	**454 ± 21**	**Ʃ Output**	**372 ± 19**
**N**_**gas**_ **= Ʃ Input - Ʃ Output**	**82 ± 38**		

Note: N_solin_ is the initial or baseline mineral N in 1.2 m soil profile sampled on 3-4-2013; N_fert_ is the N from fertilizer applied between 3-18-2013 to 6-7-2013; N_efflu_ is the N from digester effluent applied between 3-12-2013 to 3-14-2013; N_irr_ is the N in irrigation water; N_atm_ is the N from atmospheric deposition; N_crop_ is the N uptake by crop; N_solfi_ is the N left in the 1.2 m soil profile sampled on 6-7-2013; N_leach_ N lost via leaching from planting till 6-7-2013; N_gas_ is the N lost via volatilization and denitrification.

**Fig 4:**
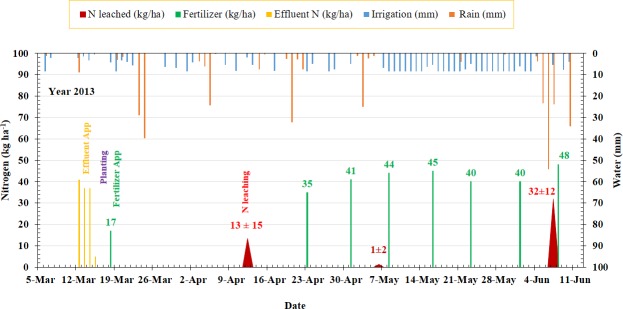
Chronosequence of leaching events in relation to water inputs (irrigation and rainfall) and nitrogen inputs (effluent N and fertilizer-N) for sweet corn grown during spring 2013.

A total of three leaching events amounting to 46.6 ± 24.4 kg ha^-1^ N were recorded during the sweet corn grown season in 2013. All leaching events were associated with the rainfall events (Fig 4). The first leaching event resulted in a N leaching loss of 13.4 ± 15.0 kg ha^-1^ N at 25 days after planting (DAP), followed by 1.1 ± 2.2 kg ha^-1^ N at 49 DAP, and a final 32.1 ± 11.5 kg ha^-1^ N loss at 81 DAP. No leaching was recorded between 49 and 81 DAP due to two reasons. First, the crop N uptake as well as water usage were at high levels during this part of the growing cycle. Bennett et al., [41] reported a period between 42 and 73 DAP as a period of linear N uptake and maximum N accumulation in aboveground biomass in corn plants. Second, the period between 49 and 78 DAP received only 5mm of rainfall and the crop ET demand was met through irrigation. Low rainfall and optimum irrigation management led to little chance for leaching. By the time rainfall arrived at 78 DAP, the phase of linear N uptake of the crop was over. However the farmer continued application of N and applied 40 and 48 kg ha^-1^ N at 75 and 82 DAP, respectively. Nitrogen applied at 75 DAP leached down to lower soil layers and finally into the lysimeters upon arrival of rainfall at 78 DAP. A total of 105 mm rainfall was received between 78 and 81 DAP which resulted in leaching loss of 32.1 ± 11.5 kg ha^-1^ N by 81 DAP. The soil cores collected to the depth of the base of the lysimeters (1.2m) on 81 DAP indicated the presence of an average 65.8 ±16.1 kg ha^-1^ mineral N in the 1.2 m soil layer. Unaware of the presence of a high amount of mineral-N in soil layer (65.8 ±16.1 kg ha^-1^N), the farmer applied an additional 48 kg ha^-1^ N (8th split application) at 82 DAP in response to the heavy rainfall to maintain the marketable quality of the sweet corn (personal communication from the farmer). This resulted in loading of soil with a total of 113.8 kg ha^-1^ N. This residual N could be vulnerable to leaching by summer rainfalls typically prevalent from May to September.

The remaining unaccounted-for N in the sweet corn N budget was significant and indicated 82.1 ± 38.2 kg ha^-1^ N lost in gaseous forms via volatilization and denitrification pathway. While it would be valuable to identify the individual N forms comprising gaseous losses as well as the time of their occurrence, determination of these losses present methodological challenges in commercial settings. Most gaseous N loss probably occurred via ammonia volatilization resulting from surface application of high pH liquid effluent before planting. This is also supported by the fact that the 1st leaching event which was recorded 25 DAP (or 28 days since last application of liquid effluent) captured only 13.4± 15.0 kg ha^-1^ N in response to 108 mm rainfall. Based on the effluent application rates, the estimated mineralizable N from liquid effluent was 121.0 kg ha^-1^ N for a 20 day period. Had there been enough mineral N (resulting from mineralization of liquid effluent) present in soil, this N would have been captured in the lysimeters in absence of a well-developed root system in young sweet corn plants. Due to the high pH of liquid effluent (>8.5), ammoniacal-N produced from ammonification of organic N, volatized quickly before it was converted to nitrate. For example, Huijsmans et al. [42] observed 70% of the total measured volatilization within 3 h after surface spreading of dairy manure. Ammonia volatilization from surface applied liquid effluents has been reported to range from 30 to 100% [42–45].

Altogether, the 2013 sweet corn N budget confirmed an N loss of 46.6 ± 24.4 kg ha^-1^ via leaching and 82.1 ± 38.2 kg ha^-1^ N via volatilization and denitrification (Table 3). The nitrogen left in soil after the crop harvest was 113.8 kg ha^-1^. In addition, an average 28.7 ±10.3 kg ha^-1^ N in crop residue remained in the field. The N lost via leaching, volatilization, and denitrification as well as potential N losses from crop residue and mineral N left in the soil sum up to a greater rates of N losses and must be acted on through adoption of best management practices such as planting a cover crop, adjusting the timing of fertilizer application and avoiding late season application of N. If crop production systems like the one presented above (2013 sweet corn) persist in the MSRB, the effectiveness of BMP’s in improving the water quality of MSRB will remain a mystery to the researchers, environmental administrators and the policy makers. The information gained from the sweet corn N budget calls for further refinement of the existing BMP’s especially with regards to use of liquids effluents and their rate and timing of application. Further, late season application of fertilizer should be discouraged among growers to reduce the N loss risk from the unused/leftover N in the soil during summer fallow period.

### Nitrogen Exports and Cycling

**Fig 5:**
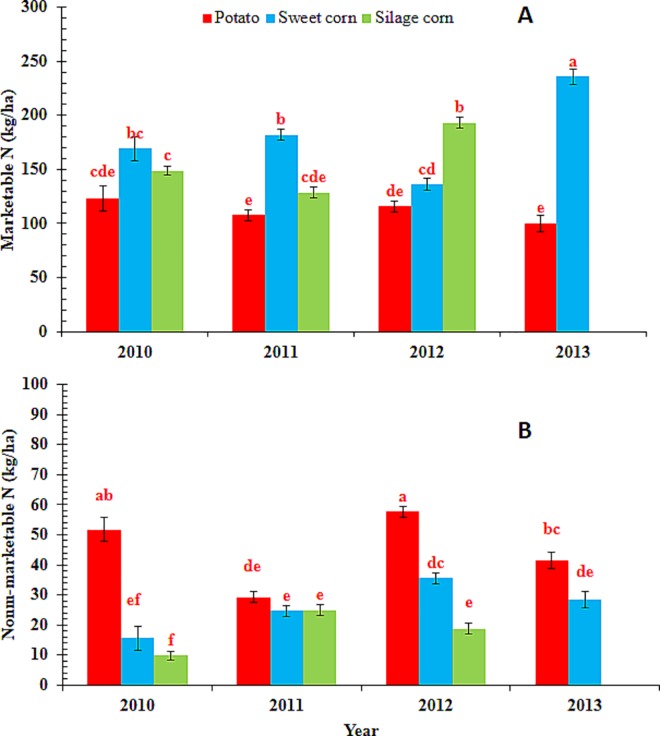
**Comparison of A) nitrogen exported off the farm in sold products (marketable N) and B) crop residue N left in the field between three crops (potato, sweet corn and silage corn) and four growing seasons (2010to 2013) at the study farm in the Middle Suwannee River Basin, Florida.** Silage corn was studied only between 2010 and 2012. Mean values of marketable N and crop residue N (represented by individual bars and their standard errors for three crops) followed by different letters indicate significant difference at α = 0.05 level.

Nitrogen present in the marketable portion of a crop (CMN) represented the amount of N exported away from the farm or sold off-farm while the N left in the non-marketable portion or crop residue (CRN) remained and recycled in the soil at the farm. Nitrogen present in tubers, ears (non-husked), stalk and leaves represent CMN while N in shoots (potato only), roots, and stubble represent the CRN. During the study period, both CMN and CRN were significantly different between years (P< 0.002; P< 0.001), and crops (P<0.0001; P< 0.05). The interaction effect of year and crop was also found significant (P<0.0001; P<0.01) (Fig 5A and 5B). Overall, the mean CMN represented 71.6%, 83.8%, and 89.0% of the mean crop N uptake and ranged from 85.5 to 142.3, 126.1 to 235.7, and 126.2 to 205.3 kg ha^-1^ N for potato, sweet corn and silage corn, respectively. The mean CRN ranged from 28.8 to 76.6, 16.5 to 41.1, and 9.8 to 29.8 kg ha^-1^ N for potato, sweet corn, and silage corn respectively. Similar estimates were found by Andraski and Bundy [38]. They reported that potato tubers consisted of 75% (or 104 kg ha^-1^ N) of the total plant N and were exported away from fields while the remaining N (34 kg ha^-1^N) was left as residue. Contrary to this, in sweet corn, only 50% of the N was removed in ears (94 kg ha^-1^ N) whereas the remaining 50% (92 kg ha^-1^ N) was left in residue and returned to soil. In the present study, the farmer exported both the sweet corn ears and aboveground plant biomass (as silage) away from the field leaving behind N in stubbles and roots in the field, which represented an average 16.2% of total plant N.

The mean CMN for tubers did not differ over the four year study period, however, significant differences were observed in CMN for sweet corn and silage corn. For silage corn, CMN was higher in 2012 compared to 2010 and 2011. Marketable crop nitrogen differed among crops in all years except 2010. In 2011, CMN was greater for sweet corn, but equal for potato and silage corn, whereas in 2012, CMN was greater for silage corn but equal for potato and sweet corn. In 2013, sweet corn had greater CMN than potato.

CRN for potato was greater than for other two crops during all year except in 2011.There was no difference in CRN between sweet and silage corn in 2010 and 2011, however, in 2012 sweet corn had a greater CRN than silage corn. Crop residues left in the field create potential for additional N loss via leaching upon their decomposition especially in the absence of a cover crop. The total dry matter left in the field in the present study amounted to 1554.1 ± 651.8, 1988.4 ± 604.7, and 2227.1 ±1433.2, kg ha^-1^, for potato, sweet corn, and silage corn, respectively. A study carried out by Bundy and Andraski [46] on irrigated sandy soils in Wisconsin Central Sand Plain found that N left in the crop residue after harvest was not recovered in the subsequent crop and was lost by leaching.

## Conclusions

Nitrogen budgets for northern Florida field crops demonstrated that fertilizer N was the primary N input for potato, sweet corn, and silage corn crops and applied at greater rates than recommended by University of Florida. A significant interaction between year and crop was observed for various pools of nitrogen. Overall, average amounts of crop N uptake and N exported away from the farm were greatest for silage corn > sweet corn > potato. Environmental N losses were greatest for potato> sweet corn > silage corn. Environmental N losses increased with increase in N inputs. Nitrogen left in crop residue was greatest for potato and posed risk for environmental N loss due to fast mineralization of potato residues. Information gained from leaching measurements from sweet corn production indicated occurrence of N leaching later in the season when the plants attained the physiological maturity. Stabilization period of lysimeters to obtain multiyear and multiple crops leaching data limited our ability to document year to year variation as well as leaching differences due to crop types- a limitation that must be thought carefully and planned meticulously when designing future studies with lysimeters. Late application of fertilizer by the farmer also resulted in accumulation of large amounts of mineral N in soil which posed high risk for leaching during the summer fallow period. Although, there is no quick fix for controlling N losses from crop production in sandy soils in the humid MSRB, management strategies to reduce N losses should focus on managing the crop residues, using recommended fertilizer rates, and avoiding late-season application of nitrogen.
